# Prostate stem cell antigen mRNA in blood is a predictor of survival after radical prostatectomy in patients with high-risk prostate cancer

**DOI:** 10.18632/oncotarget.25207

**Published:** 2018-05-29

**Authors:** Yoon Seok Suh, Jae Young Joung, Sung Han Kim, Jeong Eun Kim, Moon Kyung Choi, Weon Seo Park, Sang-Jin Lee, Ho Kyung Seo, Jinsoo Chung, Kang Hyun Lee

**Affiliations:** ^1^ Department of Urology, Center for Prostate Cancer, Hospital, National Cancer Center, Goyang, Gyeonggi-do, Korea; ^2^ Department of Pathology, Center for Prostate Cancer, Hospital, National Cancer Center, Goyang, Gyeonggi-do, Korea; ^3^ Immunotherapeutics Branch, Research Institute, National Cancer Center, Goyang, Gyeonggi-do, Korea

**Keywords:** prostate stem cell antigen, prostate cancer, biochemical recurrence, survival

## Abstract

**Objectives:**

To investigate whether the preoperative detection of prostate stem cell antigen (PSCA) mRNA in blood has predictive value for biochemical recurrence, overall survival, and cancer-specific survival after radical prostatectomy in patients with high-risk prostate cancer.

**Results:**

Median age was 67 years (interquartile range: 63-71), and median follow-up was 41 months (interquartile range: 25–65). *PSCA* mRNA was detected in 151 patients (51.1%). Biochemical recurrence was developed in 101 patients (34.2%), and all-cause mortality and prostate cancer-specific mortality occurred in 17 (5.7%) and 8 (2.7%) patients, respectively. Kaplan–Meier analysis revealed significant differences in biochemical recurrence, overall survival, and cancer-specific survival according to *PSCA* mRNA positivity. Cox regression hazards model analysis showed that *PSCA* mRNA positivity was an independent predictor of biochemical recurrence, overall survival, and cancer-specific survival.

**Conclusions:**

*PSCA* mRNA in the peripheral blood was related to poor prognosis. Detection of *PSCA* mRNA by polymerase chain reaction in peripheral blood can be used to predict survival after radical prostatectomy in patients with high-risk prostate cancer. Future study with larger cohort and long-term follow-up is required to confirm this finding.

**Materials and methods:**

A total of 295 patients with high-risk prostate cancer scheduled to undergo radical prostatectomy were prospectively enrolled from 2008 to 2016. Nested reverse transcription polymerase chain reaction was used to detect cells with *PSCA* mRNA in peripheral blood. The predicting ability of *PSCA* mRNA positivity for biochemical recurrence, overall survival, and cancer-specific survival after radical prostatectomy was evaluated.

## INTRODUCTION

Prostate cancer (PC) is the second most common cancer in men and the fifth leading cause of cancer-related death worldwide [1]. In the United States of America, PC is the second leading cause of cancer-specific deaths in men, and approximately 26,120 PC-related deaths occurred in 2016 [2]. In clinically localized PC, patients harboring high-risk disease exhibit adverse treatment outcomes [3]. Accordingly, this subset of patients is exposed to a greater risk of biochemical recurrence (BCR) or micrometastasis after radical prostatectomy (RP) compared with patients with low- or intermediate-risk disease.

The diverse management strategies for high-risk PC yield inconsistent results, representing a challenging issue in the treatment of PC. Multidisciplinary and multimodal therapeutic approaches have been applied for the management of patients with high-risk PC [4]. Of these approaches, the combination of long-term hormone therapy and external-beam radiation therapy has been accepted as a standard care for patients with high-risk PC [5–7]. In contrast, some patients with organ-confined high-risk PC remain progression-free after undergoing RP alone [8, 9]. Thus, in the era of precision medicine, selecting patients who will benefit most from RP in high-risk PC is essential.

The prostate stem cell antigen (*PSCA*) gene is located on chromosome 8q24.2, distal to c-Myc oncogene, and its amplification reported in a significant number of primary and metastatic PCs [10]. In our previous study [11], we found that *PSCA* mRNA in peripheral blood may be a good predictor of BCR in high-risk PC. In an analysis of 103 patients with high-risk disease, *PSCA* positivity in nested reverse transcription polymerase chain reaction (RT-PCR) was found to be an independent risk factor of BCR. However, sample size was limited, and the median follow-up duration was 23 months, which may have been too short for evaluation of delayed BCR development or mortality.

Accordingly, in the present study, we investigated whether the detection of *PSCA* mRNA in the blood prior to operation may have predictive value for BCR, overall survival (OS), and cancer-specific survival (CSS) after RP with in patients with high-risk PC in a long-term follow-up study.

## RESULTS

### Clinicopathological characteristics and PSCA detection by RT-PCR

In all patients, the median age was 67 years (interquartile range [IQR]: 63–71), and median follow-up duration was 41 months (IQR: 25–65). The clinicopathological characteristics are summarized in Table 1. One hundred and fifty one patients (51.1%) showed the presence of *PSCA* mRNA by RT-PCR. Overall, 30.5% of patients had prostate-specific antigen (PSA) over 20.0 ng/mL, 14.6% of patients had a biopsy Gleason score (GS) of 8–10, and 76.3% of patients had clinical stage of greater than or equal to T2c. There were no significant differences in PSA, biopsy GS, clinical stage, GS with RP specimens, extracapsular extension (ECE), seminal vesicle invasion (SVI), and surgical margin status between the two groups (*p* > 0.05).

**Table 1:** Clinicopathological characteristics of patients according to PSCA detection by RT-PCR

Variables	Number of patients (%)			*p* value
	Total, *n* = 295	RT-PCR PSCA (–), *n* = 144	RT-PCR PSCA (+), *n* = 151	
Age, years, median (IQR)	67 (63–71)	67 (62–70)	67 (63–71)	0.457
PSA (ng/mL)				0.802
<20.0	205 (69.5)	99 (68.8)	106 (70.2)	
≥20.0	90 (30.5)	45 (31.2)	45 (29.8)	
GS (biopsy)				0.997
≤7	252 (85.4)	123 (85.5)	129 (85.4)	
≥8	43 (14.6)	21 (14.5)	22 (14.6)	
Clinical stage				0.586
≤cT2b	70 (23.7)	32 (22.2)	38 (25.2)	
≥cT2c	225 (76.3)	112 (77.8)	113 (74.8)	
GS (RP specimen)				0.150
≤7	216 (73.2)	111 (77.1)	105 (69.5)	
≥8	79 (26.8)	33 (22.9)	46 (30.5)	
ECE				0.131
Positive	153 (51.9)	68 (47.2)	85 (56.3)	
Negative	142 (48.1)	76 (52.8)	66 (43.7)	
SVI				0.080
Positive	73 (24.7)	29 (20.1)	44 (29.1)	
Negative	222 (75.3)	115 (79.9)	107 (70.9)	
Surgical margin				0.375
Positive	87 (29.5)	46 (31.9)	41 (27.2)	
Negative	208 (70.5)	98 (68.1)	110 (72.8)	

Abbreviations: PSCA, prostate stem cell antigen; RT-PCR, reverse transcription polymerase chain reaction; IQR, interquartile range; PSA, prostate-specific antigen; GS, Gleason score; ECE, extracapsular extension; SVI, seminal vesicle invasion.

### BCR following RP

**Figure 1:**
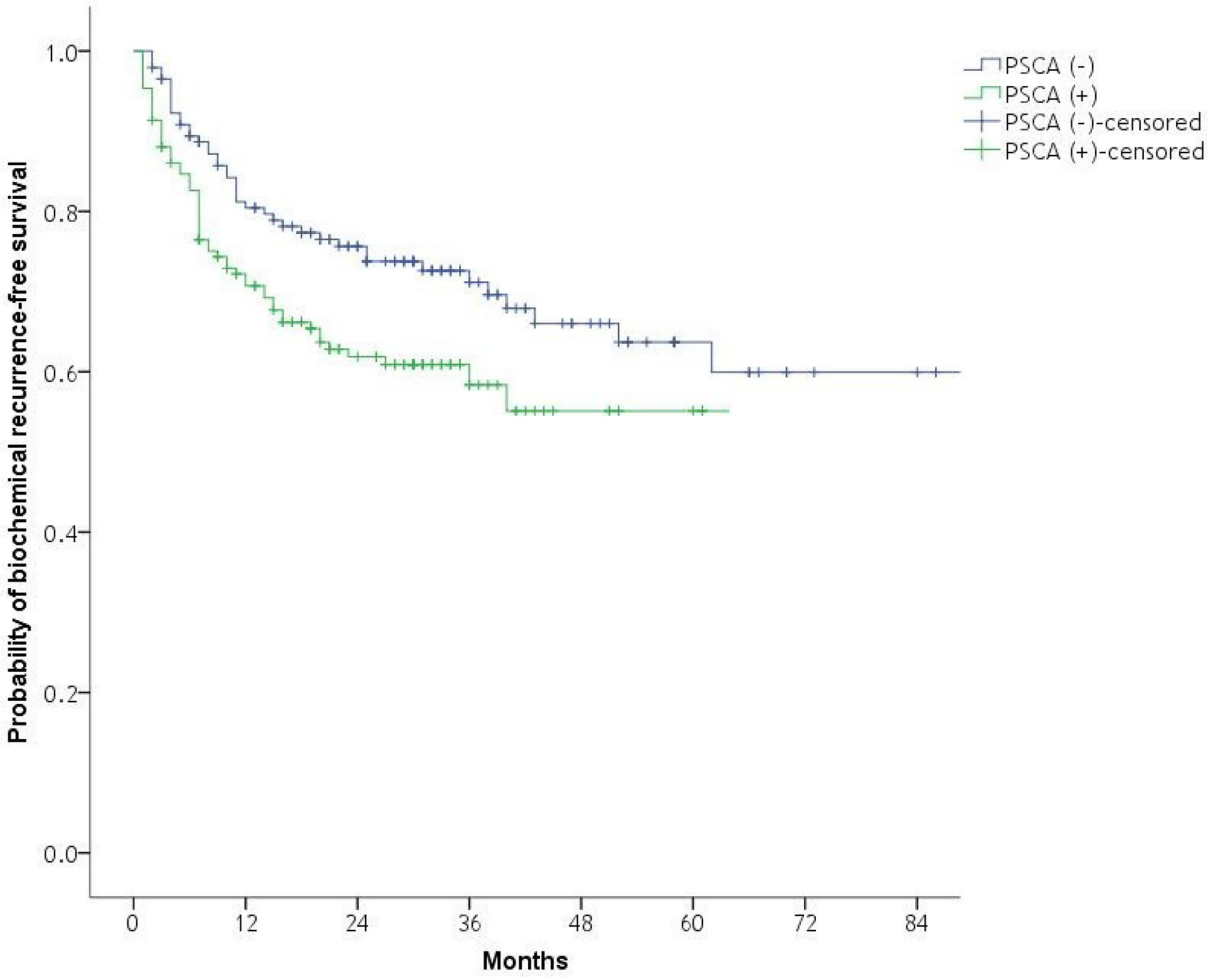
Kaplan–Meier plot of the likelihood of biochemical recurrence-free survival with respect to RT-PCR PSCA positivity after radical prostatectomy Log-rank test, *p* = 0.010. RT-PCR, reverse transcription polymerase chain reaction; PSCA, prostate stem cell antigen.

BCR was developed in 101 patients (34.2%), and the median time to BCR was 7 months (IQR: 4–15). BCR was developed in 59 patients (58.4%) with PSCA (+) and in 42 patients (41.6%) with PSCA (–). Kaplan–Meier analysis revealed a significant difference between PSCA (–) and PSCA (+) groups in BCR-free survival (Log-rank test, *p* = 0.010; Figure 1). *PSCA* mRNA positivity by RT-PCR was an independent predictor of BCR (HR: 1.817, 95% CI: 1.504–3.192, *p* < 0.001; Table 2) in Cox regression hazard analysis. PSA of greater than or equal to 20, biopsy GS of greater than or equal to 8, and pathologic stage of greater than or equal to T3 were independent predictors of BCR as well.

**Table 2:** Univariate and multivariate analyses of prognostic factors for biochemical recurrence

Variables	HR	95% CI	*p* value
Univariate			
Age	1.002	0.969–1.036	0.903
PSA ≥20	2.344	1.581–3.475	<0.001
Biopsy GS ≥8	2.626	1.718–4.012	<0.001
RT-PCR PSCA positivity	1.673	1.119–2.500	0.012
Pathologic stage ≥T3	3.844	2.435–6.174	<0.001
Multivariate			
Age			
PSA ≥20	1.576	1.043–2.382	0.031
Biopsy GS ≥8	2.247	1.632–3.931	<0.001
RT-PCR PSCA positivity	1.817	1.504–3.192	<0.001
Pathologic stage ≥T3	3.411	2.084–5.585	<0.001

Abbreviations: PSA, prostate-specific antigen; GS, Gleason score; RT-PCR, reverse transcription polymerase chain reaction; PSCA, prostate stem cell antigen.

### OS and CSS following RP

**Figure 2:**
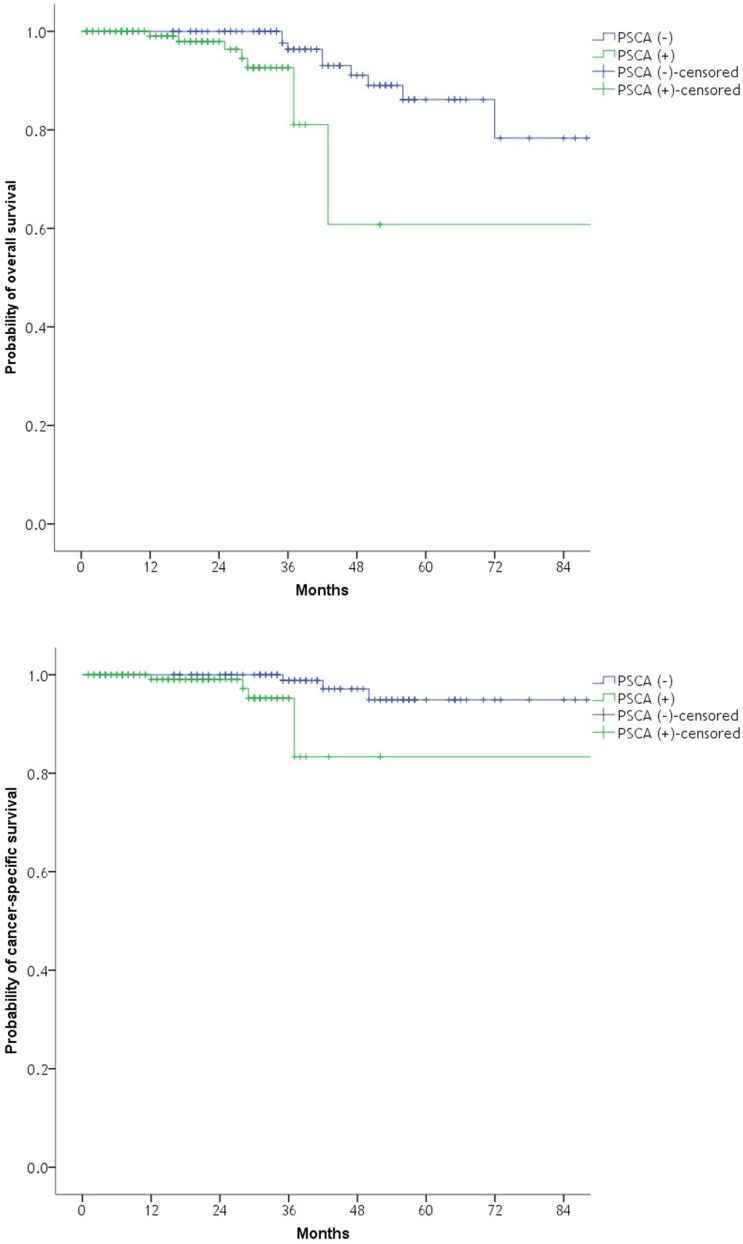
Kaplan–Meier plot of the likelihood of overall survival and cancer-specific survival with respect to RT-PCR PSCA positivity after radical prostatectomy Log-rank test, *p* = 0.004; Log-rank test, *p* = 0.014. RT-PCR, reverse transcription polymerase chain reaction; PSCA, prostate stem cell antigen.

All-cause mortality and PC-specific mortality occurred in 17 (5.7%) and 8 (2.7%) patients, respectively. Median time to all-cause mortality was 37 months (IQR: 29–49), and median time to PC-specific mortality was 36 months (IQR: 28–48). All-cause mortality occurred in eight with PSCA (+) patients (5.3%) and nine with PSCA (–) patients (6.2%). PC-specific mortality was developed in five PSCA (+) patients (3.3%) and three PSCA (–) patients (2.1%). Significant differences were found in OS and CSS with respect to RT-PCR PSCA positivity after RP in Kaplan–Meier analysis (Log-rank test, *p* = 0.004 and *p* = 0.014, respectively; Figure 2). Cox regression hazard analysis (Table 3) showed that *PSCA* mRNA positivity by RT-PCR was an independent predictor of OS (HR: 5.172, 95% CI: 1.692–15.062, *p* = 0.005) and CSS (HR: 12.784; 95% CI: 1.799–96.582; *p* = 0.008). Age was independent predictors of OS, and biopsy GS of greater than or equal to 8 was also independent predictors of OS and CSS.

**Table 3:** Univariate and multivariate analyses of prognostic factors of overall survival and cancer-specific survival

	OS			CSS		
Variables	HR	95% CI	*p* value	HR	95% CI	*p* value
Univariate						
Age	1.119	1.009–1.242	0.034	1.060	0.915–1.227	0.437
PSA ≥20	1.148	0.395–3.340	0.800	2.972	0.665–13.284	0.154
Biopsy GS ≥8	4.253	1.398–12.940	0.011	10.471	2.259–48.354	0.003
RT–PCR PSCA positivity	4.673	1.553–14.059	0.006	6.702	1.284–34.975	0.024
Pathologic stage ≥T3	2.597	1.020–8.571	0.046	8.573	1.011–72.714	0.049
Multivariate						
Age						
PSA ≥20	1.111	1.001–1.232	0.048	3.120	0.462–16.108	0.205
Biopsy GS ≥8	3.514	1.113–11.214	0.031	8.195	1.418–39.195	0.012
RT–PCR PSCA positivity	5.172	1.692–15.062	0.005	12.784	1.799–96.582	0.008
Pathologic stage ≥T3	1.910	0.674–5.182	0.314	3.392	0.411–31.856	0.274

Abbreviations: PSA, prostate–specific antigen; GS, Gleason score; RT–PCR, reverse transcription polymerase chain reaction; PSCA, prostate stem cell antigen.

## DISCUSSION

In this study, we evaluated whether the pre-operative detection of *PSCA* mRNA in blood had predictive value for BCR, OS, and CSS after RP in patients with high-risk PC. *PSCA* mRNA in blood was related to poor prognosis with regard to BCR, OS, and CSS.

Patients having high-risk PC tend to exhibit negative clinicopathological features and BCR compared with patients have low-risk disease. In previous studies, significantly higher risk of progression and PC-specific mortality was observed [12, 13]. However, although radiation therapy is recommended over RP as first-line treatment in several guidelines, there is still no evidence that RP is inferior [8]. Yossepowitch *et al.* reported that 22–63% of PC is localized high-risk disease, and 41–74% of patients remain progression-free 10 years after treatment with RP alone [12]. Thus, selecting optimal treatment for these patients is essential for achieving optimal therapeutic effects, and this issue has become increasingly important since the emergence of precision medicine. However, there are no well-established markers for predicting prognosis after initiation of therapy, including surgery, in patients with high-risk PC. Accordingly, new markers that can predict treatment outcomes with accuracy are required.

In previous reports, inconsistent results of *PSCA* mRNA detection by RT-PCR with regard to its value as a predictor of treatment outcomes have been reported [14–16]. However, analysis of these studies included heterogeneous cohorts that were relatively small. In our previous study, we reported results with 103 cases of RP using nested RT-PCR for detection of *PSCA* mRNA; our findings showed *PSCA* mRNA positivity in the peripheral blood was related to BCR after RP in high-risk disease. Another report with nested RT-PCR using peripheral blood samples in locally advanced disease demonstrated similar results [17].

In the era of precision medicine, treatment of PC will be tailored based on the characteristics of PC in each patient. Liquid biopsy using circulating tumor cells is a promising tool for assessment of response to therapies and prognosis in patients with PC. RT-PCR has also been used for highly sensitive detection of circulating tumor cells. RT-PCR for analysis of circulating tumor cells enables follow-up of biopsy analysis and is minimally invasive. Thus, RT-PCR is a suitable tool for predicting prognosis or monitoring treatment response, and liquid biopsy using RT-PCR with enhanced accuracy is urgently required for personalized treatment of PC. The present study showed that *PSCA* mRNA positivity by RT-PCR may be a useful tool for predicting treatment outcomes in patients with PC.

The present study has some limitations. Since the study was conducted with data from a single institution, selection bias may have occurred. Second, since the RP was performed by two operators, the difference of the operator might influence the outcomes. Third, with regard to the survival outcome, there was limited number of death events due to limited follow-up duration. Despite of these limitations, we believe that the findings of the present study are highly relevant because this is the study with largest cohort evaluating the value of *PSCA* mRNA in peripheral blood as a marker for predicting BCR, OS, and CSS after RP in high-risk PC to date. In addition, homogenous composition of patients in regard to treatment modality is another strength of this study. In our previous study [11], patients who received neoadjuvant hormone therapy were included, hence the possibility that neoadjuvant hormone therapy influenced PSCA expression in peripheral blood could not be excluded.

The findings of the present study suggest that *PSCA* RT-PCR could provide urologists with useful information to identify and select patients with high-risk disease who are expected to show better oncologic outcome after RP. Additionally, our results provide insights into the optimal approach for selecting patients who require adjuvant therapies, such as adjuvant radiation therapy or hormone therapy.

In conclusion, the presence of *PSCA* mRNA in peripheral blood was related to poor prognosis with regard to BCR, OS, and CSS. Detection of *PSCA* mRNA by RT-PCR in peripheral blood can be used to predict survival after RP in patients with high-risk PC. Future study with larger cohort and long-term follow-up is required to confirm this finding.

## MATERIALS AND METHODS

### Ethics statement

This study was approved by the Institutional Review Board (IRB) of our institution (IRB approval number: NCCNCS05049). All patient records were anonymized and de-identified before analysis. The study protocol was consistent with the ethical guidelines of the World Medical Association Declaration of Helsinki Ethical Principles for Medical Research Involving Human Subjects. Informed consent was obtained from all subjects for the collection of clinicopathological data, serum samples, and tissue samples.

### Subjects

A total of 295 patients with high-risk PC who were scheduled to undergo RP were prospectively enrolled from 2008 to 2016. Eligible patients had histologically proven prostate adenocarcinoma. High-risk PC was defined according to the presence of any one of the following criteria: clinical stage ≥ T2c, serum PSA ≥ 20 ng/mL, or biopsy GS ≥ 8 [3]. All patients underwent RP and standard pelvic lymph node dissection. RP was performed by two urologists (KHL and JYJ).

### Blood samples, extraction of RNA, and reverse transcription

Peripheral blood sampling was performed pre-operatively. After Ficoll-Plaque plus gradient centrifugation (GE Healthcare, Little Chalfont, UK), nucleated cell fractions were separated from 5 mL whole blood samples that were anticoagulated with ethylenediaminetetraacetic acid (EDTA). Extraction of RNA and reverse transcription were conducted as performed in our prior report [18].

### PCR and nested PCR

Primers were designed by Bioneer (Daejeon, Republic of Korea). The intron spanning primer pairs that were specific for human PSCA were as follows: sense, 5′-TGCTTGCCCTGTTGATGGCAG-3′, and antisense, 5′-ACGTGAGCCGGACGACGAGAC-3′. The antisense primer was replaced by 5′-TACTCCTGCAAAGCCCAGGT-3′ for nested PCR. The housekeeping gene β-actin was used as an internal control. PCR was conducted for 25 cycles in a volume of 20 μL containing 1 mM sense and antisense primers, 1 mL RT product, 10 mM Tris-HCL (pH 9.0), 1.5 mM MgCl_2_, 0.25 mM of each dNTP, 30 mM KCl, and 1 U Taq-DNA polymerase (Bioneer). For amplifying cDNA (0.5 mg), a tube-controlled thermal cycler (MJ Research, Waltham, MN, USA) was used. Amplification of PCR products (2 mL) was performed for additional 40 cycles utilizing the same reagents for nested PCR, although nested primers replaced the original primers.

### Sensitivity of RT-PCR assays and PCR product analysis

The sensitivity of the RT-PCR assay was determined by measuring LNCaP human PC cell dilutions in peripheral blood mononuclear cell (PBMC) suspensions. RT-PCR assay sensitivity was determined as described in our previous study [18]. It was found that the mRNAs of 10 LNCaP cells which was diluted in 10^7^ PBMCs from healthy donors could be detected by nested RT-PCR for PSCA. Samples that were subjected to qualitative RT-PCR for detection of *PSCA* mRNA were analyzed several times to confirm the reproducibility of the test. After the initial results were confirmed by re-assay, results for each sample were submitted for subsequent analyses. For PCR product analysis, a premix of 10 μL PCR product with loading dye was evaluated using a PCR kit (Bioneer). Subsequently, samples were loaded onto agarose gels (2%) in TBE buffer (0.1 M Tris [pH 8.4], 90 mM boric acid, 1 mM EDTA). The samples were then subjected to electrophoresis for 30 min. Staining of gels was performed using ethidium bromide, and visualization of amplicons was carried out with an ultraviolet transilluminator. To confirm internal consistency, scoring of assay results was performed by two investigators (WSP and MKC) who were blinded to any clinicopathological information.

### Pathological analysis

Complete transverse sectioning was performed from the base to the apex of prostrate specimens with 4-mm intervals. Prostate specimens were examined by a single pathologist (WSP) and staged using the TNM system (International Union Against Cancer). GSs, the presence of ECE and SVI were evaluated.

### Follow-up

All 295 patients were scheduled to undergo serum PSA evaluation with routine blood tests and routine checkups that included history taking and physical examination, such as digital rectal examination, every 3 months for the first 2 years after RP, biennially from the third to the fifth years, and then annually thereafter. Imaging evaluations using computed tomography scanning, magnetic resonance imaging, and bone scanning were evaluated if clinically indicated. BCR was defined as a sustained elevation of PSA more than 0.4 ng/mL on at least two occasions. Clinical outcome analyses were performed using the NCC PC database.

### Statistical analysis

Independent-sample *t*-tests were used for between-group comparisons. Fisher’s exact test was used to compare categorical data. The Kaplan–Meier method was used to evaluate survival outcomes, and the log-rank test was used to assess differences. Univariate and multivariate analyses were conducted with the Cox proportional hazards regression model. For deriving a final model of variables with independent significant relationships with survival, variables with *p* values of less than 0.20 in univariate analysis were included in multivariate analysis. All statistical analyses were performed using SPSS software for Windows (version 20; SPSS, Chicago, IL, USA). A *p* value of less than 0.05 was considered statistically significant, and all statistical tests were two-sided.

## Abbreviations

*PSCA*: prostate stem cell antigen
PC: prostate cancer
BCR: biochemical recurrence
RP: radical prostatectomy
RT-PCR: reverse transcription polymerase chain reaction
HR: hazard ratio
CI: confidence interval
OS: overall survival
CSS: cancer-specific survival
IQR: interquartile range
PSA: prostate-specific antigen
GS: Gleason score
ECE: extracapsular extension
SVI: seminal vesicle invasion
